# Long-term survival after esophagectomy with distal pancreatectomy for locally advanced esophageal cancer with pancreatic invasion: a case report

**DOI:** 10.1186/s40792-021-01338-w

**Published:** 2021-12-14

**Authors:** Yoshiki Kaneko, Katsuji Hisakura, Koichi Ogawa, Yoshimasa Akashi, Yusuke Ohara, Yohei Owada, Tsuyoshi Enomoto, Kinji Furuya, Shoko Moue, Manami Doi, Kazuhiro Takahashi, Osamu Shimomura, Shinji Hashimoto, Noriaki Sakamoto, Tsunehiko Maruyama, Tatsuya Oda

**Affiliations:** 1 Department of Gastrointestinal and Hepato-Biliary-Pancreatic Surgery, University of Tsukuba, 1-1-1, Tennoudai, Tsukuba, Ibaraki 305-8575 Japan; 2 Department of Surgery, Mito Saiseikai General Hospital, 3-3-10, Futabadai, Mito, Ibaraki 311-4145 Japan; 3 Department of Pathology, University of Tsukuba, 1-1-1, Tennoudai, Tsukuba, Ibaraki 305-8575 Japan

**Keywords:** Esophagectomy, Pancreatectomy, Locally advanced esophageal cancer, Pancreatic invasion

## Abstract

**Background:**

The treatment for the locally advanced esophageal cancer invading adjacent organs is controversial. We performed a radical surgery for a patient suffering from lower esophageal cancer with pancreatic invasion, and led to long-term survival.

**Case presentation:**

A 62-year-old man with dysphagia, was endoscopically diagnosed lower esophageal cancer. Abdominal computed tomography shows that the tumor formed a mass with the solitary metastatic abdominal lymph node, which invaded pancreas body and gastric body. He was diagnosed locally advanced esophageal cancer cStage IIIC. As chemoradiotherapy was difficult because of the high risk of gastric mucosal damage, radical esophagectomy with distal pancreatectomy and reconstruction of gastric conduit were performed. The postoperative course was uneventful and the patient was discharged 16 days after operation. At present, 7 years after surgery, he is still alive with disease-free condition.

**Conclusion:**

Esophagectomy with distal pancreatectomy may be feasible for locally advanced esophageal cancer with pancreatic invasion in terms of curability and long-term survival.

## Background

Esophageal cancer has a high-grade malignant potential, and many patients are diagnosed with far-advanced cancer [1, 2]; the lack of serosa in the esophagus facilitates invasion. The depth of tumors invading adjacent structures, such as aorta, vertebral body, and trachea is defined as T4, generally. T4 esophageal cancer results in a poor survival, even after treatment [2–4]. On the other hand, pancreatic invasion of esophageal cancer is uncommon, and is not clearly defined as T4.

The most effective treatment modality for T4 esophageal cancer is chemoradiotherapy (CRT) [5]. However, CRT for lower esophageal cancer is attended with difficulty because of the high risk of gastric mucosal damage associated with irradiation [6]. To our knowledge, there are few articles on T4 esophageal cancer invading pancreas, where esophagectomy with distal pancreatectomy has been performed to describe its usability for the long-term survival. Here, we describe a case that lower esophageal cancer with pancreatic invasion, underwent esophagectomy and distal pancreatectomy, and we suggest the possibility for survival benefit.

## Case presentation

**Fig. 1:**
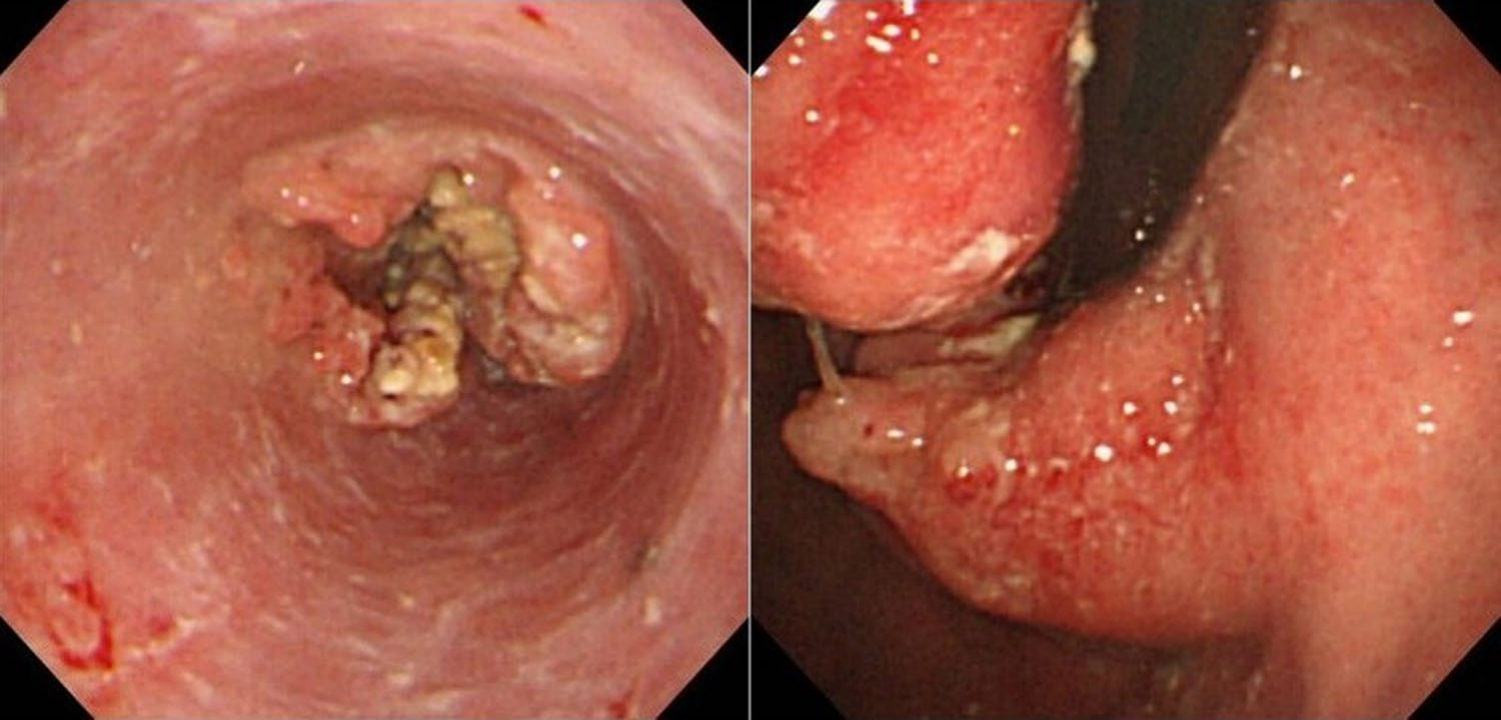
Esophagogastroduodenoscopy revealed the circumferential type2 lesion of lower esophagus (35–45 cm from the incisors). Only small caliber scope was allowed to through the lesion due to severe stenosis. The lesion directly invaded to gastric fundus

**Fig. 2:**
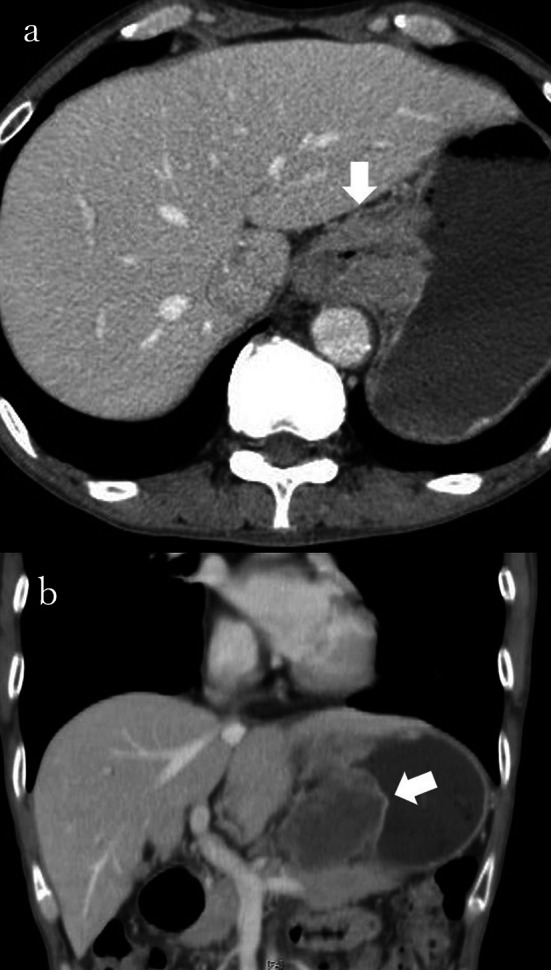
Findings of computed tomography. **a** Thickened wall as primary lesion was observed from the lower esophagus to fundus of stomach (arrow). **b** A solitary metastatic abdominal lymph node measuring 56 mm made a mass and invaded to lesser curvature and pancreatic body (arrow)

A 62-year-old man, affected dysphagia, was endoscopically diagnosed with lower esophageal cancer confirmed squamous cell carcinoma on biopsy, and was reffered to our hospital (Fig. 1). He had no medical history. His lifestyle has included 1500 ml beer consumption per day and 40 cigarettes per day for the past 40 years. Computed tomography (CT) showed thickening of the wall in the lower esophagus as the primary lesion was demonstrated and the tumor formed a mass with the solitary metastatic abdominal lymph node, and invaded pancreas body and gastric body (Fig. 2). No other distant metastasis was detected on CT. He was diagnosed with lower esophageal cancer cT4 N1 M0, with pancreatic invasion, cStage IIIC according to 7th edition of the Union for International Cancer Control system [7]. At first, we considered definitive chemoradiotherapy. However, radiation oncologists evaluated that the tumor was less candidate for chemoradiotherapy because of the risk of gastric mucosal damage. For the purpose of definitive therapy, radical esophagectomy with distal pancreatectomy was planned. As neoadjuvant chemotherapy, CF therapy (cisplatin and 5-fluorouracil therapy; cisplatin was dripped 80 mg/m^2^ plus 5-fluorouracil was infused 800 mg/m^2^ on day 1 through 4 continuously) was started according to standard therapy of localized advanced esophageal cancer [8]. However, after once administration, he could not continue chemotherapy for the exacerbation of dysphagia, and underwent radical surgery. Preoperative evaluation of tumor was similar to initial findings on endoscopy and CT.

**Fig. 3:**
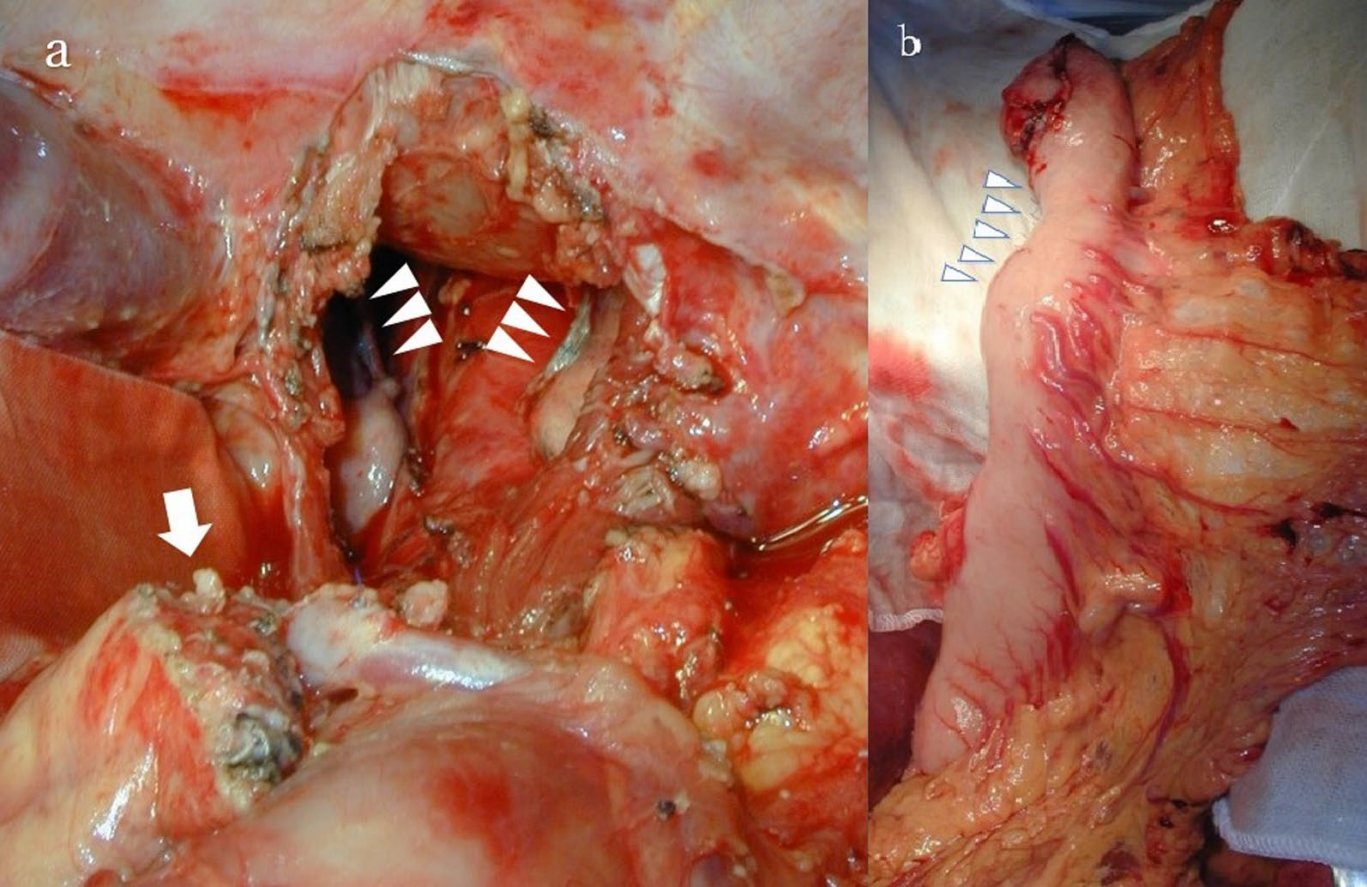
Findings after resection. **a** Cut end of pancreas was revealed at the ventral side of splenic vein at the junction of infra mesenteric vain (arrow). The bilateral pulmonary ligaments were also resected, because primary tumor invasion was suspected. Descending aorta, epicardium and bilateral lung was observed through esophageal hiatus (arrow heads: the cut end of bilateral pulmonary ligaments). **b** The gastric conduit. The lesser curvature of gastric body was resected, because the metastatic lymph node was invaded (arrow heads)

In findings on laparotomy, abdominal lymph node was infiltrated directly to pancreas body. As the radical surgery, Ivor Lewis esophagectomy with distal pancreatectomy and splenectomy, followed by reconstruction of gastric conduit. Two fields lymphadenectomy was performed according to the treatment strategy of the abdominal esophageal cancer. Reconstruction of gastric conduit was possible although the lymph node was adherent to lesser side of gastric body, which was resected when reconstruction of gastric conduit. In addition, partial resection of lung was performed simultaneously owing to involvement of bilateral pulmonary ligaments to the primary tumor (Fig. 3). The operative time was 528 min. The estimated blood loss was 2850 ml, and the patient was transfused 4 units of red blood cell concentrates.

The patient had an uneventful postoperative course and was able to take orally. He was discharged 16 days after operation with tube-free. After discharge, he underwent two courses of CF therapy as adjuvant chemotherapy. At present, he is still alive and has no recurrence for 7 years after surgery.

**Fig.4:**
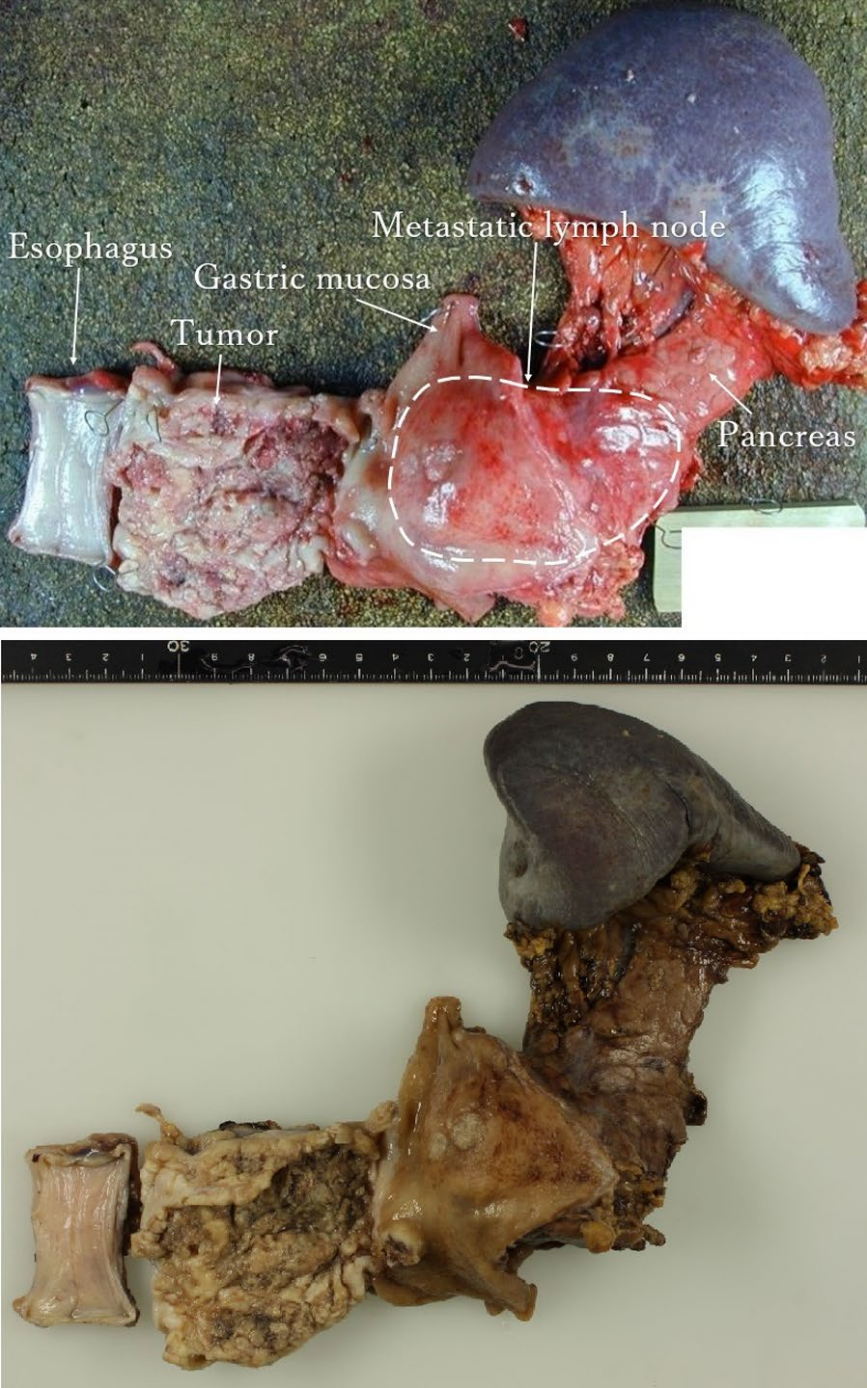
Resected specimen findings. Primary lesion was observed from lower esophagus to esophagogastric junction (type 3 tumor, measuring 75 × 55 × 15 mm in size). Metastatic lymph node was fixed to stomach and pancreas (measuring 70 × 50 × 40 mm in size)

**Fig.5:**
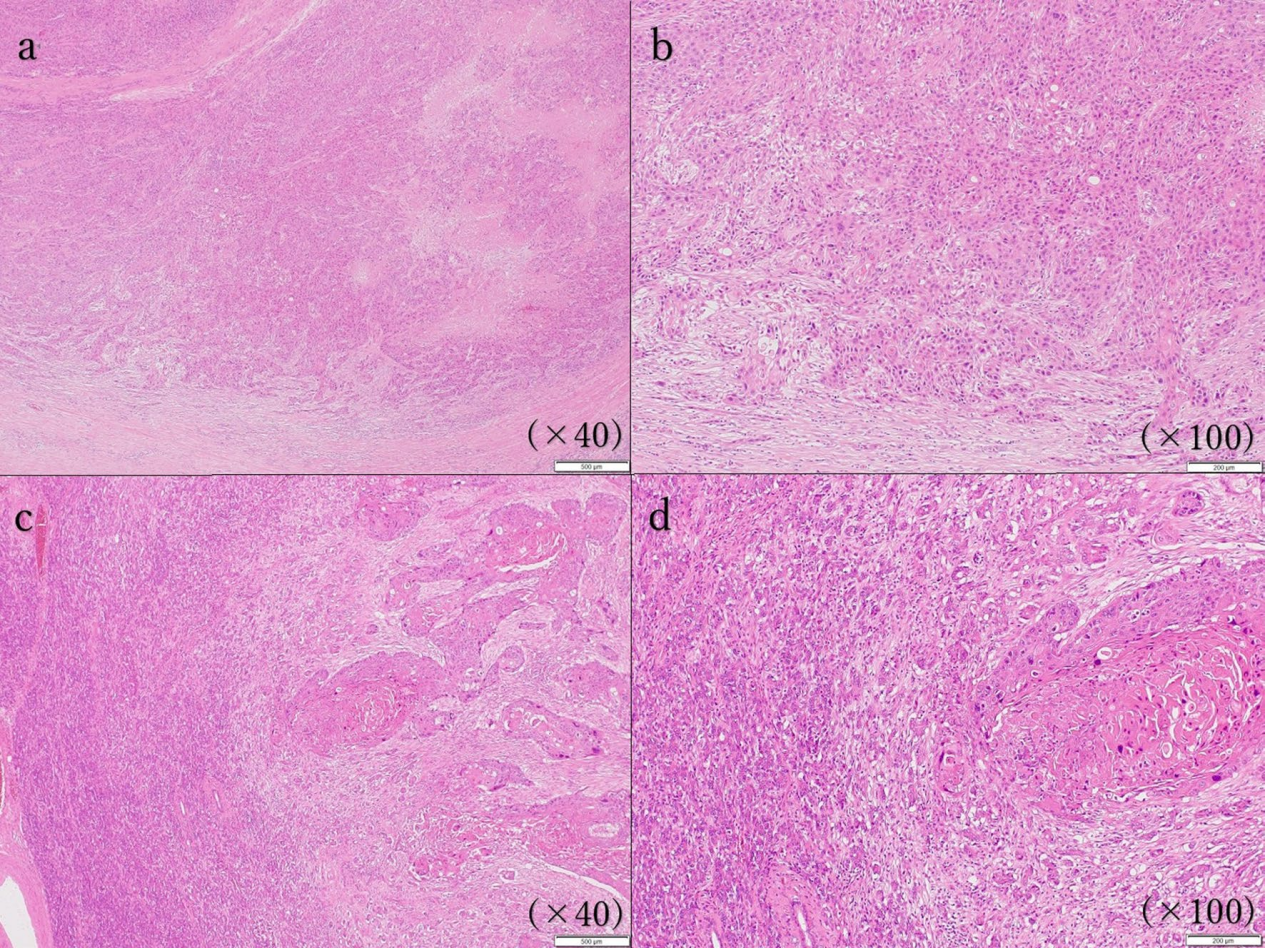
Histopathological findings (Hematoxylin and eosin staining). **a**, **b** Esophagus and primary lesion. Tumor invaded adventitia, but not adjacent organs. **c**, **d** Pancreas and metastatic lymph node. The infiltration of metastatic lymph node into pancreas was observed

In the resected specimen, the primary lesion was observed from lower esophagus to esophagogastric junction, and metastatic lymph node was fixed to stomach and pancreas body (Fig. 4). Pathological examination revealed that primary lesion was not infiltrated to lung (Fig. 5a, b). Massive metastatic lymph node (over 5cm) was observed in the lesser curvature of stomach, and infiltrated to pancreas and gastric wall with extranodal extension (Fig. 5c, d). The tumor was diagnosed with squamous cell carcinoma, moderately differentiated type. The vascular and lymphatic invasion was confirmed. The surgical margin was negative. The pathological stage was ypT4 N1 (1/61) M0 (metastatic lymph node invasion into pancreas), ypStage IIIC. The histopathological response of chemotherapy was grade 1a, which was equivalent that proliferable cells were 2/3 or more, in 7th edition of the Union for International Cancer Control system [7].

## Discussion

In this study, the patient who had locally advanced lower esophageal cancer with pancreatic invasion was able to receive radical esophagectomy with distal pancreatectomy safely, and achieved long-term survival with no recurrence. To the best of our knowledge, this is the first report to suggest the oncological predominance of radical surgery (esophagectomy with distal pancreatectomy) for esophageal cancer invading pancreas.

Prognostic outcome of esophagectomy with distal pancreatectomy for the patient with locally advanced esophageal cancer is unclear because of few literatures. The outcome of T4 esophageal cancer naturally differs among the invaded organs. For example, the tumors invading trachea and/or bronchus (classified as T4b) are relatively common and have poor prognosis [9]. The literature describing the prognosis of patients who underwent esophagectomy with pancreatectomy is summarize in Table 1 [10–14]. Long-term survival was observed in 2/6 (33.3%) cases. No literature has referred the prognostic effect of pancreatic invasion in esophageal cancer. In case of gastric cancer, gastrectomy with partial pancreatectomy might be considered as a valid curative treatment option when the tumor is directly invading into pancreas [15]. In case of colon cancer, Marsman et al. reported that post-operative cumulative survival was 100% in 8 patients performed colectomy with pancreatectomy at the end of follow-up (median 24 months [95% CI 9–111]) [16]. Direct pancreatic invasion of cancer may have no negative factors in cancer prognosis. On the other hand, Saito et al. referred that solitary lymph node metastasis and absence of upper mediastinal lymph node metastasis contributed to long-term survival [12]. Some literatures reported that the number of lymph node metastasis was the prognostic factor after radical esophagectomy [17–19]. In this case, only one lymph node metastasis was found in 61 dissected lymph nodes, pathologically.

**Table 1:** Reported cases of esophagectomy with distal pancreatectomy for locally advanced esophageal cancer

Age, Sex	Reason (pancreatectomy)	Operative procedure/reconstruction method	Reccurence	Prognosis	Report (year)
53, Male	Primary tumor invaded	Left thoraco-laparotomic inferior esophagectomy Total gastrectomy, Distal pancreatectomy Lateral segmentectomy of the liver/Roux-en Y	ND (Lymph node)	10 M (alive)	Matsubara et al. (2003)
62, Male	Metastatic tumor (stomach) invaded	Right thoraco-laparotomic lower esophagectomy, total gastrectomy, distal pancreatectomy/Roux-en Y	10 M (Lymph node)	16 M (dead)	Hata et al. (2007)
52, Male	Metastatic lymph node ivaded	Right thoraco-laparotomic subtotal esophagectomy Distal pancreatectomy/gastric conduit (postmediastinal route)	None	84 M (alive)	Saito et al. (2011)
59, Male	Metastatic tumor (stomach) invaded	Right thoraco-laparotomic subtotal esophagectomy Partial gastrectomy, Left lateral segmentectomy of liver Distal pancreatectomy/gastric conduit	ND (Pleural dissemination)	6 M (dead)	Nakazawa et al. (2012)
64, Male	Metastatic lymph node ivaded	Right thoraco-laparotomic subtotal esophagectomy Distal pancreatectomy/Roux-en Y and gastric conduit	7 M (Liver)	18 M (dead)	Nishiwaki et al. (2018)
62, Male	Metastatic lymph node ivaded	Right thoraco-laparotomic subtotal esophagectomy Distal pancreatectomy/gastric conduit (postmediastinal route)	None	84 M (alive)	Our case

CRT has been considered one of the treatment options, though no standard treatment for T4 esophageal cancer has been established [2, 20]. However, the tumor in this case has spread from lower esophagus to stomach, which is likely to be damaged by irradiation [6]. On the other hand, esophagectomy combined with perioperative chemotherapy is also standard therapy for resectable locally advanced esophageal cancer [8, 21]. In this case, the tumor was evaluated as resectable by esophagectomy with distal pancreatectomy, and was undergone preoperative chemotherapy, followed by radical surgery.

A suitable treatment strategy for T4 cases has not been established and remains unclear, as well as extent of lymphadenectomy, which have the potential to raise surgical stress and complication rate. In our case, two field lymph node dissection including mediastinal lymph nodes was performed as a part of radical surgery, even though no metastatic lymph node was diagnosed pathologically in the mediastinum. Typical two- or three-field lymph node dissection including prophylactic dissection is recommended for T4 esophageal cancer because of a better prognosis in esophagectomy [22]. In this literature review, there was a recurrent case of mediastinal lymph node after esophagectomy with Appleby surgery, in which mediastinal dissection was not performed [11]. If possible, typical lymph node dissection may be favorable.

Neoadjuvant therapy for advanced esophageal cancer is also controversial. In this case, neoadjuvant chemotherapy was performed, although only once administration due to the adverse event. A current standard treatment modality for patients with resectable esophageal cancer is neoadjuvant chemotherapy followed by radical surgery (with prophylactic two- or three-field lymphadenectomy) [8]. On the other hand, neoadjuvant CRT is accepted as the standard treatment for locally advanced esophageal cancer in Western countries [23, 24]. However, neoadjuvant CRT is found to be associated with increased mortality and morbidity after surgery due to irradiation toxicity [25, 26]. Therefore, our strategy in this case might reduce the therapeutic toxicity and lead to no major complications, consequently. Further study is necessary to examine the survival benefit and the toxicity by the neoadjuvant chemotherapy or CRT for esophageal cancer, in particular lower esophagus, which is easily damaged with irradiation, and is accessible to pancreas. A randomized controlled trial is ongoing by Japanese Clinical Oncology Group, comparing CF therapy versus DCF therapy (cisplatin, 5-fluorouracil, and docetaxel therapy) versus CF-RT (cisplatin, 5-fluorouracil and radiation therapy) as neoadjuvant treatment for locally advanced esophageal cancer [27], and this study could be possible to apply to the benefit and toxicity of CF-RT to the esophageal cancer with pancreatic invasion.

## Conclusion

We present a patient who was suffered from lower esophageal cancer with infiltration into pancreas and stomach, was performed esophagectomy with distal pancreatectomy after neoadjuvant chemotherapy according to standard therapy of T2–3 localized advanced esophageal cancer, and led to long-term survival. Esophagectomy with distal pancreatectomy for pancreatic invasion of esophageal cancer is worthful to perform for curability.

## Abbreviations

CRT: Chemoradiotherapy
CT: Computed tomography
CF therapy: Cisplatin and 5-fluorouracil therapy
DCF therapy: Cisplatin, 5-fluorouracil, and docetaxel therapy
CF-RT: Cisplatin, 5-fluorouracil and radiation therapy
